# Fibromyalgia-related costs and loss of productivity: a substantial societal burden

**DOI:** 10.1186/s12891-016-1027-6

**Published:** 2016-04-16

**Authors:** Anaïs Lacasse, Patricia Bourgault, Manon Choinière

**Affiliations:** Département des sciences de la santé, Université du Québec en Abitibi-Témiscamingue, Rouyn-Noranda, Québec Canada; Centre de recherche du Centre hospitalier universitaire de Sherbrooke, Sherbrooke, Québec Canada; École des sciences infirmières, Faculté de médecine et des sciences de la santé, Université de Sherbrooke, Sherbrooke, Québec Canada; Département d’anesthésiologie, Faculté de médecine, Université de Montréal, Montréal, Québec Canada; Centre de recherche du Centre hospitalier de l’Université de Montréal, Tour Saint-Antoine 850, rue Saint-Denis, Bureau S03-428, Montréal, Québec Canada

**Keywords:** Cost of illness, Direct costs, Productivity loss, Fibromyalgia, Chronic pain, Societal costs

## Abstract

**Background:**

This study aimed at describing pain-related health care resource use, direct costs, and productivity loss among patients suffering from fibromyalgia syndrome (FMS).

**Methods:**

A cost-of-illness study with a sample of 57 adults having a diagnosis of FMS was conducted in the province of Quebec (Canada). Data regarding FMS-related direct costs and productivity loss from paid and unpaid work over a three-month period were collected using a standardized structured telephone interview protocol. Direct costs were valued in 2009 Canadian dollars using a societal perspective.

**Results:**

Results showed that average direct costs over a three-month period added up to $951 per patient (SD: $710), which could be translated in a mean annual cost of $3804. The purchase of prescribed medications led to the highest costs (mean: $329, SD: $321), followed by consultations to health care professionals other than physicians (mean: $129, SD: $222) and physicians consultations (mean: $98, SD: $116). Results further showed a high economic burden for patients themselves, aside from costs covered by public or private insurers. Among the subsample of participants who had a paid job (45.6 %), an average of 5.6 days (SD: 13.2) were lost due to pain during the past three months. Among those who were not employed (54.4 %), an average of 25.1 days in household productivity (SD: 24.8) were lost.

**Conclusions:**

FMS is associated with a substantial socioeconomic burden. Further research is clearly needed to improve the management of this type of disorder and make better decisions regarding resource allocation.

**Electronic supplementary material:**

The online version of this article (doi:10.1186/s12891-016-1027-6) contains supplementary material, which is available to authorized users.

## Background

Fibromyalgia syndrome (FMS) is a chronic disorder with key symptoms such as chronic widespread musculoskeletal pain, sleep disturbances, cognitive dysfunctions, mood disturbances and fatigue [1]. A number of studies have estimated the prevalence of FMS to be as high as 4.9 % in women and 2.9 % in men [1–5]. FMS has substantial impacts on the physical functioning, the mental health and the quality of life of those suffering from it [6–8] as well as their spouses [9]. A review of the current literature describing the relationships between FMS, physical and mental health-related quality of life reported that such patients had significantly lower physical and mental health scores as compared to the general population or patients suffering from other types of chronic pain (*p* < 0.05) [10], suggesting that FMS represents a major burden on those suffering from it.

As part of the complete assessment of a disease burden on patients and society, cost-of-illness studies are necessary to inform the public, the health care system, the third party payers as well as the policy makers of the socioeconomic impact of health disorders [11–13]. Such health economic studies can consider direct costs, defined as medical care expenditures and non-medical costs associated with the provision of medical services (e.g. transportation to and from health care institutions), and productivity costs (time and productivity losses from paid and unpaid work due to the illness) [11, 13]. To be comprehensive, the perspective of the cost analysis should be societal, i.e. all costs should be considered no matter if the expenses are incurred by the patients, the public health care system or private thirds party insurers [14].

Many published studies have evaluated the economic burden of FMS [15–36]. For instance, using an United-States health-insurance claim database, Berger and colleagues [15] found that FMS patients had annual direct medical costs that were approximately three times higher (mean: US$9573, SD: US$20,135) than patients without FMS (mean: US$3291, SD: US$13,643) that were matched for age and gender (*n* = 33,176 in each group). Many other studies found a heavier economic burden of FMS compared with other disorders or healthy subjects [17, 19, 20, 23, 26, 28, 29, 31, 34, 35] while others studies have not found significant differences [18, 33, 36]. However, these cost-of-illness studies are not easily comparable for a number of reasons such as the perspective of the cost analysis, the health care system particularities varying per country and the cost components considered. In fact, many of these studies failed to use a societal perspective and to consider a number of components of care who are not necessarily covered by health insurance plans such as costs of over-the-counter medications, complementary alternative medicines, medical aids, and out-of-pocket expenses associated with traveling to and from health care institutions. Furthermore, the measurement of productivity loss as much for employed FMS patients as for unemployed FMS patients was not measured in many of these studies, even though it is highly important in order to recognize the real societal burden of an illness [37].

Few FMS cost-of-illness studies have been conducted in Canada [20, 24, 35], which has the particularity of having a publicly funded health care system, universal coverage for medically necessary health care services and coverage for prescription drugs among individuals who do not have access to a private drug insurance program [38, 39]. For example, a study conducted by Lachaine and colleagues [20] compared the health care utilization and costs among FMS patients and a control group without FMS (*n* = 16,010 in each group). Their study used data from the Quebec administrative databases (health and prescription drug claims) and focused on a restricted number of components of care such as physician visits, physician’s interventions, prescription drugs and hospitalizations. Their results suggested a higher annual economic burden for FMS ($4065, SD: $6798) as compared to the control group ($2766, SD: $5945). White and colleagues [35] reached similar conclusions when adopting the perspective of the Ontario public health insurance program. Although informative, these studies failed to consider a number of components of care who are not covered by public health insurance plans. Until now, only one Canadian study reported a societal cost analysis among FMS patients [24]. However, their study did not include costs associated with seeking medical care which underestimates FMS-related direct non-medical costs.

The present study thus aimed at describing pain-related health care resource use, direct medical and non-medical costs, and productivity loss among FMS patients. It is believed that this study will help to increase the recognition of the burden that FMS has on society and can eventually contribute to decision making for better resource allocation.

## Methods

### Study design

The present cost-of-illness study used a cross-sectional design with a sample of FMS patients who were about to start to participate in a randomized controlled trial (RCT) on the efficacy of a multimodal structured interdisciplinary group intervention for the self-management of FMS called the PASSAGE Program (French acronym for *Programme d’Apprentissage de StratégieS d’Auto-Gestion Efficaces*) [40].

### Study population

Study participants recruitment was conducted in two university-affiliated settings of the province of Quebec (Canada) in September 2009: 1) Sherbrooke, a large city located in the southeastern area of the province, and 2) Rouyn-Noranda, a small city located in the Abitibi-Témiscamingue region located in the northwestern part of the province. The local Ethics Committees of the *Centre hospitalier universitaire de Sherbrooke* and the *Université du Québec en Abitibi-Témiscamingue* reviewed and approved the research protocol and the patient consent form. Recruitment was conducted through announcements in local newspapers in both cities encouraging individuals with FMS to contact the research team. To be eligible, participants had to be at least 18 years old, had to have a medical diagnosis of FMS based on the American College of Rheumatology (ACR) classification criteria [41] for at least 6 months, had to report pain levels of at least moderate intensity (≥4/10) in the seven days prior to enrolment, and finally they had to be motivated to participate in the PASSAGE Program RCT. Exclusion criteria were being pregnant or lactating, suffering from an active cancer, uncontrolled metabolic disease or other major physical or psychiatric disorder, and having an outstanding litigation regarding claim for disability payments. Specific details about the PASSAGE Program, the eligibility assessment, and the recruitment of participants are described elsewhere [40]. Each patient gave informed consent before inclusion in the study.

### Data collection methods

Standardized structured telephone interviews (see Additional file 1) were completed among study participants alongside the baseline evaluation of the PASSAGE Program RCT. These interviews were conducted by well-trained research assistants in order to gather data about FMS-related health care resource use, direct medical and non-medical costs, and productivity loss during the past three months. A 3-month recall period was shown to be the ideal time frame to maximise the validity of self-reported health care resources use [42]. Telephone interviews were favored over self-administered questionnaires due to the complexity of economic data collection.

### Measurement of direct costs

This study measured the economic burden of FMS from a societal perspective, i.e. all costs were considered no matter who pays the expenses [14]. Specifically, participants were asked to report details about the number of hospitalizations, emergency department (ED) visits, and all types of physician or other health care professionals they had consulted for the management of FMS pain symptoms in the previous three months. Data related to prescribed and over-the-counter FMS medications, natural health products, and medical aids purchased were also collected. Finally, costs related to paid at-home help and other costs related to FMS management (e.g., aquafit classes, travel and parking fees related to medical appointments) were collected. Specific details about these different costs components, different payers and information used for direct medical and non-medical costs valuation are presented in Table 1. For each component, costs were calculated by multiplying the number of occasions when a health care resource was reported to be used by the unitary costs of the resource (see Additional file 2). Direct costs associated with the management of FMS were all calculated in Canadian dollars (CAD) for the year 2009.

**Table 1:** Cost valuation of FMS-related components of care

Components of care	Payers in the context of the province of Quebec	Monetary valuation method and sources of unitary costs^a^
Hospitalizations	Provincial public health insurance	Self-reported number of visits were multiplied by the mean cost of an hospitalization/ED visit obtained from Health and Social Services Centres of Sherbrooke and Rouyn-Noranda
ED visits		
Physician consultations	Provincial public health insurance	Self-reported number of visits were multiplied by unitary costs of a visit retrieved from the *Régie de l’assurance maladie du Québec* Fee manual for general practitioners and specialists
Consultations to other health care professionals	Variable^b^	Self-reported number of visits and associated self-reported out-of-pocket expenses
Hospital, ED, and healthcare visits (Travelling)	Patients	Self-reported distances (kilometers) were valued using *Revenu Quebec’s* Rates applicable to the use of an automobile. When applicable, costs were estimated using the Quebec government’s Compendium of Tariffs of Private Transportation by Taxi
Hospital, ED, and health care visits (Parking)	Patients	Self-reported out-of-pocket expenses
Over-the-counter medications	Patients	Self-reported out-of-pocket expenses
Prescribed medications	Provincial public prescription drug insurance or private drug insurers	Self-reported medication use was valued using costs published in the *Régie de l’assurance maladie du Québec* Prescription drug list of medications
Natural health products	Patients	Self-reported out-of-pocket expenses
Medical aids	Generally the patients^c^	Self-reported out-of-pocket expenses
Employment of domestic help due to health status impairment	Patients	Self-reported out-of-pocket expenses
FMS-related additional expenses		

^a^All unitary prices represent 2009 values

^b^For the majority of health care professionals (e.g. massage therapists, acupuncturists, chiropractors, private domain physiotherapists), patients pay the bill with a possibility of reimbursement if they have a private health insurance. Some consultations (e.g. nurses, pharmacists, public domain physiotherapists) are not charged to the patients and are included in the public health care or community pharmacies services. Those costs were not considered in the analysis

^c^ With a possibility of reimbursement by the public or private insurer in some cases

*FMS* Fibromyalgia syndrome, *ED* Emergency department

### Measurement of productivity loss

As part of the standardized structured telephone interviews, workers and non-workers were asked to report the number of days that were lost (*During the past 3 months, how many days have you been absent from work/have you had to cease your caregiving and household unpaid work because of your pain or pain-related medical visits?).* Time and productivity costs were not estimated in our study due to the complexity associated with calculating reliable estimates of the monetary value of loss of productivity [43].

### Data analysis

Descriptive statistics such as frequency tables, means, standard deviations (SD), medians, minimums, and maximums were calculated to summarize health care resources use, costs for each component of care, total costs, as well as time lost from unpaid and paid work. Statistical analysis were performed using SAS version 9.2 (SAS Institute, Cary, NC, USA).

## Results

A total of 57 FMS patients completed the telephone interview (32 from the region of Sherbrooke and 25 from the region of Rouyn-Noranda). The sample was mostly composed of female patients (92.98 %). The mean age of study participants was 48.41 years old (SD = 10.43) and more than half of them (54.39 %) did not have a paid job in the three months preceding the interview.

Table 2 shows the frequencies of health care resource use during the past three months. A small proportion of study participants reported FMS-related hospitalizations (1.75 %) and ED visits (10.53 %). FMS-related visits to a physician were more frequent with 70.17 % of participants reporting at least one visit in the past three months. Family practitioners were the type of physicians the most often consulted. Visits to other health care professionals were also frequent with 64.91 % of participants reporting at least one visit in the past three months. The most frequently consulted health care professionals other than physicians were massage therapists, acupuncturists, chiropractors, pharmacists, and physiotherapists. Five participants (8.77 %) reported at least one FMS-related medical intervention or test in the past three months (Table 2).

**Table 2:** FMS-related health care resource utilization during the past 3 months

Health care resources (n = 57)	n	(%)
Hospitalizations		
Number of hospitalizations (max: 1)		
0	56	98.25
1	1	1.75
ED visits		
Number of ED visits (max: 3)		
0	51	89.47
1	4	7.02
2 or more	2	3.51
Physician consultations		
Number of physician consultations (max: 6)		
0	17	29.82
1	23	40.35
2 or more	17	29.82
Family physician (max: 6)	36	63.16
Anaesthetist (max: 1)	2	3.51
Neurosurgeon (max: 1)	2	3.51
Neurologist (max: 1)	3	5.26
Prosthetist-orthotist (max: 1)	1	1.75
Physiatrist (max: 1)	1	1.75
Psychiatrist (max: 1)	3	5.26
Rheumatologist (max: 1)	3	5.26
Gastroenterologist (max: 2)	2	3.51
Respirologist (max: 1)	1	1.75
Urologist (max: 1)	1	1.75
Consultations to other health care professionals^a^		
Number of consultations to other health care professionals (max: 22)		
0	20	35.09
1 to 4	20	35.09
5 or more	17	29.82
Number of medical interventions and tests^b^		
0	52	91.23
1	2	3.51
2 or more	3	5.26
Over-the-counter medications purchase^c^ (max: 5)		
Yes	38	66.67
Prescribed medications purchase^d^ (max: 13)		
Yes	51	89.47
Natural health products purchase (max: 6)		
Yes	30	52.63
Medical aids purchase^e^ (max: 4)		
Yes	12	21.05
Employment of domestic help due to health status impairment		
Yes	9	15.79
FMS-related additional expenses^f^		
Yes	16	28.07

Notes:

^a^Including nurse, acupuncturist, chiropractor, kinesiologist, massage therapist, naturopath, osteopath, pharmacist, physiotherapist, psychoeducator, psychologist, sexologist, and social worker

^b^Including gastroscopy, injection of corticosteroids, and magnetic resonance

^c^Including acetaminophen, acetylsalicylic acid, analgesic rubs, antiacids, collagen products, ibuprofen, laxatives, and muscle relaxants

^d^Including 28:08 Analgesics and antipyretics (includes nonsteroidal anti- inflammatory agents and opiate agonists), 28:12 Anticonvulsants, 28:16 Psychotropics (includes antidepressants and antipsychotic agents), 28:20 Central nervous system stimulants, 28:24 Anxiolytics, sedatives and hypnotics, 28:32 Antimigraine agents, 28:36 Antiparkinsonian Agents,12:20 Skeletal muscle relaxants, 52:16 Local anesthetics, 56:12 Cathartics and Laxatives, 56:22 Antiemetics, 56:28 Antiulcer agents and acid suppressants, 56:32 Prokinetic agents, 08:30.08 Antimalarials, 24:24 Bêta-adrenergics blocking agents, 24:28 Calcium-channel blocking agents, 24:32 Renin-angiotensin system inhibitors, 40:28 Diuretics, and 92:00 Miscellaneous Therapeutic Agents according to the American Hospital Formulary Service (AHFS) classification system

^e^Including bathroom aids, bedroom specialized equipment, canes, heat/cold bags, heating electric pads, heating patches, lumbar support, orthosis, strapping/taping bandages, and transcutaneous electrical nerve stimulation (TENS) devices and accessories

^f^Including accommodations for outside town medical visits, ambulance fees, exercise/relaxation classes, house renovations (installation of custom medical equipment), and fees related to the reproduction/transmission of medical charts

*FMS* Fibromyalgia syndrome, *ED* Emergency department

Regarding pain treatment modalities, 66.67 % of study participants purchased over-the counter medications, 89.47 % prescribed medications, and 52.63 % natural health products. Other medical aids expenses such as bathroom aids, bedroom specialized equipment, canes, heat/cold bags, heating electric pads, heating patches, lumbar support, orthesis, strapping/taping bandages, and transcutaneous electrical nerve stimulation (TENS) devices/accessories were also incurred by one in five participants (21.05 %). Up to 15.79 % of participants had a paid domestic help due to their health status. Finally, other FMS-related costs such as travel expenses to go to health care appointments and hotel accommodation fees for outside of town medical visits, ambulance fees, exercise/relaxation classes, house renovations (installation of custom medical equipment) and fees related to the reproduction/transmission of medical charts were incurred by 28.07 % of participants (Table 2).

Table 3 shows the direct FMS-related costs during the three months preceding the interviews. The total mean cost added up to $950.51 per patient (SD: $710.09; median: $767.82). Prescribed FMS-related medications represented the biggest expenses (mean: $329.38; SD: $321.13; median: $234.00) followed by consultations to health care professionals other than physicians (mean: $129.19; SD: $221.53; median: $50.00) and physician visits (mean: $97.57; SD: $116.40; median: $51.80). No statistically significant difference in FMS-related total costs was found between patients recruited in the region of Sherbrooke vs Rouyn-Noranda (*p* = 0.9551).

**Table 3:** FMS-related direct costs during the past 3 months

	Costs per patient ($)					Payers in the context of the province of Quebec		
Expenses (n = 57)	Mean ± SD		median	min	max	Public insurance	Private insurance	Patient out-of-pocket expenses
Hospitalizations	14.35	± 108.35	0	0	818.00	x		
ED visits	24.32	± 81.23	0	0	462.00	x		
Physician consultations	97.57	± 116.40	51.80	0	488.50	x		
Consultations to other health care professionals	129.19	± 221.53	50.00	0	1 240.00		x	x
Hospital, ED, and health care visits (Travelling)	62.41	± 98.17	21.45	0	467.58			x
Hospital, ED, and health care visits (Parking)	2.40	± 3.86	0	0	17.00			x
Over-the-counter medications	27.97	± 34.86	14.00	0	155.00			x
Prescribed medications	329.38	± 321.13	234.00	0	1 471.61	x	x	
Natural health products	63.67	± 228.58	10.00	0	1 620.00			x
Medical aids	23.32	± 74.47	0	0	450.00			x
Employment of domestic help due to health status impairment	72.32	± 200.91	0	0	1 080.00			x
FMS-related additional expenses	38.81	114.97	0	0	700.00			x
Total cost	950.51	710.09	767.82	0	3 014.94			

*FMS* Fibromyalgia syndrome, *ED* Emergency department

Table 4 shows the total number of days lost from paid and unpaid work in the previous three months. Of the 26 working participants, an average number of days of 5.59 (SD: 13.18; median: 1.75) was lost due to FMS. Of the 31 non-working participants, an average number of days of 25.09 (SD: 24.77; median: 25.00) was lost.

**Table 4:** FMS-related productivity loss during the past 3 months

Time lost from paid work among the 26 working patients (days)	
mean ± SD:	5.59 ± 13.18
median:	1.75
min:	0
max:	65.50
Time lost from unpaid work among the 31 non-working patients (days)	
mean ± SD:	25.09 ± 24.77
median:	25.00
min:	0
max:	85.00

*FMS* Fibromyalgia syndrome, *SD* standard deviation

## Discussion

The present study aimed at describing the societal economic burden of FMS. Direct medical and non-medical costs and productivity loss data were gathered from a sample of FMS patients from two regions of the province of Quebec (Canada). As of now, very few studies estimated the economic burden of FMS in Quebec [20] and elsewhere in Canada [24, 35]. The results of the present study suggest that FMS has a significant economic burden on those suffering from it as well as society in general which includes the public health care system and third party payers.

In our study, the average 3-month direct societal cost of FMS was found to be $951 per patient with fairly high variability (SD: $710; range: $0–$3015). This suggests an extrapolated annual direct cost of $3804 CAD per patient (range: $0–$12,060). Consistent with our findings, Penrod and colleagues [24] adopted a Canadian societal perspective and estimated that FMS was associated with an average 6-month direct cost of $2298 (which could be translated in a $4596 annual direct cost per patient). As for other Canadian studies, Lachaine and colleagues [20] estimated that from the perspective of the Quebec public health care system, FMS was associated with an average annual direct cost of $4065 per patient. When adopting the Ontario public health insurance program perspective, White and colleagues [35] reported that the average annual economic burden of FMS was $1028 per patient. These variations in results can be explained by the variability of costing methods that were used (e.g. perspective, cost components considered), the year the study was conducted, and the study samples characteristics (e.g., patients from core urban areas can have a better access to specialized health care services which could result in higher costs as compared to patients of rural areas).

In the present sample, the highest direct costs were for prescribed FMS-related medications, which are covered in vast majority by public or private insurances in the province of Quebec. Nearly every participant (89 %) reported having purchased prescribed FMS-related medications in the previous three months and some patients bought up to 13 different prescription drugs. It is noteworthy to mention that above and beyond the purchase of prescribed medications, 67 % also purchased over-the-counter pain medication and 53 % purchased natural health products which represent patient out-of-pocket costs. The second highest costs were for consultations to health care professionals other than physicians (65 % of patients reported such costs), which are often incurred by patients as well. Our results underline the high economic burden of FMS for patients themselves aside from costs covered by public or private insurers. Consistent with these findings, another Canadian study reported that chronic non-cancer pain patients’ health-related out-of-pocket expenses exceeded costs covered by public insurance or private third party insurers [44].

There is currently no cure for FMS and management of this disorder is aimed at reducing symptoms and maintaining optimal functioning [45, 46]. Interventions such as medication alone produce, at best, modest effects on patients’ condition [47, 48]. Moreover, it was shown that non-pharmacological treatments are more effective than drug interventions in FMS [49]. The promotion of multimodal treatment approaches among FMS patients is highly recommended [50–53] but our study underlines the substantial out-of-pocket expenses for patients seeking different healthcare treatments. This situation raises questions in the context of a health care system claimed to offer universal coverage for medically necessary health care services provided on the basis of need rather than the ability to pay [38]. This issue sketches future research avenues about the costs and benefits of patient self-seeking multimodal treatment vs turnkey multimodal self-management programs that could be offered by health care centers for the management of FMS.

In terms of productivity loss caused by FMS, the present study showed that, among the subsample of patients who were employed, an average of 5.59 days of work was lost due to pain, which could be translated in 3.19 weeks annually. Even non-working patients experienced non-negligible losses in household productivity as a result of chronic pain (average of 25.09 days of unpaid work in the past three months which could be translated in 14.34 weeks annually). Although no monetary values were assigned to the FMS-related productivity loss due to the complexity associated with calculating reliable estimates [43], the results are indicative once again of a significant FMS burden on society. In fact, previous research has shown that productivity costs account for most of the economic burden of FMS [24, 25]. Although we cannot make any assumption about the causal relationship between FMS and unemployment using our data, the present study highlights the magnitude of unemployment in the FMS population with only 46 % of the present sample being employed. These results are in line with another Canadian study which reported that only 42 % of FMS women were working and that time lost from paid work because of FMS was approximatively 4 weeks annually [24].

### Strengths and limitations

The present study has a number of strengths and limitations that should be highlighted. First, data was gathered through standardized and structured telephone interviews by trained research assistants. Second, the cost-of-illness analysis used a societal perspective. Instead of only focusing on a restricted number of components of care as it was done in Canadian studies described earlier [20, 35], the present study assessed multiple components from ED visits and healthcare professionals consultations to the purchase of medical aids and the hiring of domestic help. However, because some unitary costs were self-reported by participants, data was vulnerable to a recall bias (e.g., costs of over-the-counter medication and costs of visits to healthcare professionals other than physicians such as massage therapists). This bias was however minimized by using a short recall period [42]. Future cost-of-illness studies could circumvent this limitation by the linkage between administrative databases (for the estimation of costs covered by the health care system) and patient reported data (for the estimation of costs covered by private third party insurers and out-of-pocket expenses). In our study, no unitary costs were assigned to productivity loss which may have led to an underestimation of the true full societal costs of FMS. Furthermore, the small sample size may limit the precision of our estimates and the generalizability of the present study. However, the proportion of women (93 %) and the mean age of patients (48 years) in our study population were comparable to what was reported in other samples of FMS patients: Women: 68 to 93 % [15, 20, 35, 54], Mean age : 46 to 59 years [15, 20, 24, 54]. The fact that patients were recruited from two different regions of the province of Quebec also increases the external validity of the present results.

## Conclusions

In spite of their shortcomings, cost-of-illness studies are being relied on increasingly to inform both public and private decision-makers regarding the expenditures related to diseases and injuries. These studies help drive decisions about future insurance benefits, research efforts in curbing and controlling diseases and injuries, and development of programs to improve the health of the population [37]. The present results suggest a significant societal economic burden of FMS. However, management of chronic pain is currently believed to be sub-optimal. In fact, several studies have shown that this condition is underdeclared, underrecognized, underdiagnosed, and inadequately treated in medical practice [55–60]. The present results can help raise decision-makers’ and healthcare professionals’ awareness which could eventually lead to more training, resource allocation as well as better access to multimodal self-management programs that could help alleviate the burden of FMS on patients and society.

### Ethics and consent to participate

The local Ethics Committees of the *Centre hospitalier universitaire de Sherbrooke* and the *Université du Québec en Abitibi-Témiscamingue* reviewed and approved the research protocol and the patient consent form. Each patient gave informed consent before inclusion in the study.

### Consent to publish

Not applicable.

## Additional files

Additional file 1: Interview protocol. (PDF 571 kb) Additional file 2: Dataset. (XLSX 49 kb)

## Abbreviations

ACR: American College of Rheumatology
CAD: Canadian dollars
FMS: fibromyalgia syndrome
RCT: randomized controlled trial
SD: standard deviation
TENS: transcutaneous electrical nerve stimulation

## References

[1] Wolfe F, Clauw DJ, Fitzcharles MA, Goldenberg DL, Katz RS, Mease P, Russell AS, Russell IJ, Winfield JB, Yunus MB (2010). The American College of Rheumatology preliminary diagnostic criteria for fibromyalgia and measurement of symptom severity. Arthritis Care Res.

[2] Gran JT (2003). The epidemiology of chronic generalized musculoskeletal pain. Best Pract Res Clin Rheumatol.

[3] Branco JC, Bannwarth B, Failde I, Abello Carbonell J, Blotman F, Spaeth M, Saraiva F, Nacci F, Thomas E, Caubere JP (2010). Prevalence of fibromyalgia: a survey in five European countries. Semin Arthritis Rheum.

[4] McGillion MH, Watt-Watson J, Stevens B, Lefort SM, Coyte P, Graham A (2008). Randomized controlled trial of a psychoeducation program for the self-management of chronic cardiac pain. J Pain Symptom Manage.

[5] White KP, Speechley M, Harth M, Ostbye T (1999). The London Fibromyalgia Epidemiology Study: the prevalence of fibromyalgia syndrome in London, Ontario. J Rheumatol.

[6] Da Costa D, Dobkin PL, Fitzcharles M, Fortin PR, Beaulieu A, Zummer M, Senecal J, Goulet JR, Rich E, Choquette D (2000). Determinants of health status in fibromyalgia: a comparative study with systemic lupus erythematosus. J Rheumatol.

[7] Bennett RM, Schein J, Kosinski MR, Hewitt DJ, Jordan DM, Rosenthal NR (2005). Impact of fibromyalgia pain on health‐related quality of life before and after treatment with tramadol/acetaminophen. Arthritis Care Res.

[8] Oliveira PJ, Costa ME (2013). Relationships Among Affect, Emotional Distress, and Physical Health Status in Fibromyalgia. J Autoimmun Dis Rheumatol.

[9] Bigatti SM, Cronan TA (2002). An examination of the physical health, health care use, and psychological well-being of spouses of people with fibromyalgia syndrome. Health Psychol.

[10] Hoffman D, Dukes E (2008). The health status burden of people with fibromyalgia: a review of studies that assessed health status with the SF‐36 or the SF‐12. Int J Clin Pract.

[11] Berger ML, Bingefors K, Heldblom EC, Pashos CL, Torrance GW, Dix SM (2010). Health care cost, quality, and outcomes: ISPOR Book of terms.

[12] Larg A, Moss J (2011). Cost-of-illness studies: a guide to critical evaluation. Pharmacoeconomics.

[13] Tarricone R (2006). Cost-of-illness analysis. What room in health economics?. Health Policy.

[14] Neumann PJ (2009). Costing and perspective in published cost-effectiveness analysis. Med Care.

[15] Berger A, Dukes E, Martin S, Edelsberg J, Oster G (2007). Characteristics and healthcare costs of patients with fibromyalgia syndrome. Int J Clin Pract.

[16] Berger A, Sadosky A, Dukes EM, Edelsberg J, Zlateva G, Oster G (2010). Patterns of healthcare utilization and cost in patients with newly diagnosed fibromyalgia. Am J Manag Care.

[17] Boonen A, van den Heuvel R, van Tubergen A, Goossens M, Severens J, van der Heijde D, van der Linden S (2005). Large differences in cost of illness and wellbeing between patients with fibromyalgia, chronic low back pain, or ankylosing spondylitis. Ann Rheum Dis.

[18] Doron Y, Peleg R, Peleg A, Neumann L, Buskila D (2004). The clinical and economic burden of fibromyalgia compared with diabetes mellitus and hypertension among Bedouin women in the Negev. Fam Pract.

[19] Kleinman N, Harnett J, Melkonian A, Lynch W, Kaplan-Machlis B, Silverman SL (2009). Burden of fibromyalgia and comparisons with osteoarthritis in the workforce. J Occup Environ Med.

[20] Lachaine J, Beauchemin C, Landry P-A (2010). Clinical and economic characteristics of patients with fibromyalgia syndrome. Clin J Pain.

[21] Lind BK, Lafferty WE, Tyree PT, Diehr PK, Grembowski DE (2007). Use of complementary and alternative medicine providers by fibromyalgia patients under insurance coverage. Arthritis Care Res.

[22] Sanchez R, Uribe C, Li H, Alvir J, Deminski M, Chandran A, Palacio A (2011). Longitudinal evaluation of health care utilization and costs during the first three years after a new diagnosis of fibromyalgia. Curr Med Res Opin.

[23] Palacio A, Uribe CL, Li H, Hanna J, Deminski M, Alvir J, Chandran A, Sanchez R (2010). Financial and clinical characteristics of fibromyalgia: a case–control comparison. Am J Manag Care.

[24] Penrod JR, Bernatsky S, Adam V, Baron M, Dayan N, Dobkin PL (2004). Health services costs and their determinants in women with fibromyalgia. J Rheumatol.

[25] Rivera J, Rejas J, Esteve-Vives J, Vallejo MA (2009). Resource utilisation and health care costs in patients diagnosed with fibromyalgia in Spain. Clin Exp Rheumatol.

[26] Robinson RL, Birnbaum HG, Morley MA, Sisitsky T, Greenberg PE, Claxton AJ (2003). Economic cost and epidemiological characteristics of patients with fibromyalgia claims. J Rheumatol.

[27] Robinson RL, Birnbaum HG, Morley MA, Sisitsky T, Greenberg PE, Wolfe F (2004). Depression and fibromyalgia: treatment and cost when diagnosed separately or concurrently. J Rheumatol.

[28] Sicras-Mainar A, Rejas J, Navarro R, Blanca M, Morcillo Á, Larios R, Velasco S, Villaroya C (2009). Treating patients with fibromyalgia in primary care settings under routine medical practice: a claim database cost and burden of illness study. Arthritis Res Ther.

[29] Thompson JM, Luedtke CA, Oh TH, Shah ND, Long KH, King S, Branda M, Swanson R (2011). Direct medical costs in patients with fibromyalgia: cost of illness and impact of a brief multidisciplinary treatment program. Am J Phys Med Rehabil.

[30] Wassem R, Hendrix TJ (2003). Direct and indirect costs of fibromyalgia to patients and their families. J Orthop Nurs.

[31] White LA, Birnbaum HG, Kaltenboeck A, Tang J, Mallett D, Robinson RL (2008). Employees with fibromyalgia: medical comorbidity, healthcare costs, and work loss. J Occup Environ Med.

[32] Wolfe F, Anderson J, Harkness D, Bennett RM, Caro XJ, Goldenberg DL, Russell IJ, Yunus MB (1997). A prospective, longitudinal, multicenter study of service utilization and costs in fibromyalgia. Arthritis Rheum.

[33] Santoro MS, Cronan TA (2013). Depression, self-efficacy, health status, and health care costs: A comparison of men with fibromyalgia or osteoarthritis. J Musculoskelet Pain.

[34] Jeffery DD, Bulathsinhala L, Kroc M, Dorris J (2014). Prevalence, Health Care Utilization, and Costs of Fibromyalgia, Irritable Bowel, and Chronic Fatigue Syndromes in the Military Health System, 2006–2010. Mil Med.

[35] White KP, Speechley M, Harth M, Ostbye T (1999). The London fibromyalgia epidemiology study: Direct health care costs of fibromyalgia syndrome in London, Canada. J Rheumatol.

[36] Silverman S, Dukes EM, Johnston SS, Brandenburg NA, Sadosky A, Huse DM (2009). The economic burden of fibromyalgia: Comparative analysis with rheumatoid arthritis. Curr Med Res Opin.

[37] Clabaugh G, Ward MM (2008). Cost‐of‐Illness Studies in the United States: A Systematic Review of Methodologies Used for Direct Cost. Value Health.

[38] Health Canada. Canada’s Health Care System. http://www.hc-sc.gc.ca/hcs-sss/pubs/system-regime/2011-hcs-sss/index-eng.php. Accessed 26 March 2015

[39] Régie de l’assurance maladie du Québec (2012). Rapport annuel de gestion 2011–2012.

[40] Bourgault P, Lacasse A, Marchand S, Courtemanche-Harel R, Charest J, Gaumond I, BarcellosdeSouza J, Choiniere M (2015). Multicomponent interdisciplinary group intervention for self-management of fibromyalgia: a mixed-methods randomized controlled trial. PLoS ONE.

[41] Wolfe F, Smythe HA, Yunus MB, Bennett RM, Bombardier C, Goldenberg DL, Tugwell P, Campbell SM, Abeles M, Clark P (1990). The American College of Rheumatology 1990 Criteria for the Classification of Fibromyalgia. Report of the Multicenter Criteria Committee. Arthritis Rheum.

[42] Bhandari A, Wagner T (2006). Self-reported utilization of health care services: improving measurement and accuracy. Med Care Res Rev.

[43] Lensberg BR, Drummond MF, Danchenko N, Despiegel N, Francois C (2013). Challenges in measuring and valuing productivity costs, and their relevance in mood disorders. Clinicoecon Outcomes Res.

[44] Guerriere DN, Choiniere M, Dion D, Peng P, Stafford-Coyte E, Zagorski B, Banner R, Barton PM, Boulanger A, Clark AJ (2010). The Canadian STOP-PAIN project - Part 2: What is the cost of pain for patients on waitlists of multidisciplinary pain treatment facilities?. Can J Anaesth.

[45] Fitzcharles M-A, Ste-Marie PA, Goldenberg DL, Pereira JX, Abbey S, Choiniere M, Ko G, Moulin DE, Panopalis P, Proulx J (2012). 2012 Canadian Guidelines for the diagnosis and management of fibromyalgia syndrome: Executive summary. Pain Res Manag.

[46] Fitzcharles MA, Ste-Marie PA, Goldenberg DL, Pereira JX, Abbey S, Choiniere M, Ko G, Moulin DE, Panopalis P, Proulx J (2013). Canadian Pain Society and Canadian Rheumatology Association recommendations for rational care of persons with fibromyalgia. A summary report. J Rheumatol.

[47] Han C, Lee SJ, Lee SY, Seo HJ, Wang SM, Park MH, Patkar AA, Koh J, Masand PS, Pae CU (2011). Available therapies and current management of fibromyalgia: focusing on pharmacological agents. Drugs Today (Barcelona, Spain: 1998).

[48] Sim J, Adams N (2002). Systematic review of randomized controlled trials of nonpharmacological interventions for fibromyalgia. Clin J Pain.

[49] Rossy LA, Buckelew SP, Dorr N, Hagglund KJ, Thayer JF, McIntosh MJ, Hewett JE, Johnson JC (1999). A meta-analysis of fibromyalgia treatment interventions. Ann Behav Med.

[50] Argoff CE, Albrecht P, Irving G, Rice F (2009). Multimodal analgesia for chronic pain: rationale and future directions. Pain Med.

[51] American Society of Anesthesiologists Task Force on Chronic Pain Management & American Society of Regional Anesthesia and Pain Medicine (2010). Practice guidelines for chronic pain management: An updated report by the American Society of Anesthesiologists Task Force on Chronic Pain Management and the American Society of Regional Anesthesia and Pain Medicine. Anesthesiology.

[52] Sommer C. Fibromyalgia: A Clinical Update. Pain. 2010;XVIII(4):1-4.

[53] Turk DC, Swanson KS, Tunks ER (2008). Psychological approaches in the treatment of chronic pain patients--when pills, scalpels, and needles are not enough. Can J Psychiatry.

[54] Vij B, Whipple MO, Tepper SJ, Mohabbat AB, Stillman M, Vincent A (2015). Frequency of Migraine Headaches in Patients With Fibromyalgia. Headache.

[55] Zuccaro SM, Vellucci R, Sarzi-Puttini P, Cherubino P, Labianca R, Fornasari D (2012). Barriers to pain management: focus on opioid therapy. Clin Drug Investig.

[56] MacDonald NE, Flegel K, Hebert PC, Stanbrook MB (2011). Better management of chronic pain care for all. CMAJ.

[57] AETMIS (2006). Prise en charge de la douleur chronique (non cancéreuse): Organisation des services de santé.

[58] Kingma EM, Rosmalen JG (2012). The power of longitudinal population-based studies for investigating the etiology of chronic widespread pain. Pain.

[59] Sessle BJ (2012). The pain crisis: what it is and what can be done. Pain Res Treat.

[60] Arnold LM, Clauw DJ, McCarberg BH, FibroCollaborative (2011). Improving the recognition and diagnosis of fibromyalgia. Mayo Clin Proc.

