# Supplementary material for: Fibromyalgia-related costs and loss of productivity: a substantial societal burden
Source: BMC Musculoskelet Disord. 2016 Apr 16;17:168. doi: 10.1186/s12891-016-1027-6 (PMC4833946; doi:10.1186/s12891-016-1027-6)
Supplement: Additional file 1: — Interview protocol. (PDF 571 kb) [file 12891_2016_1027_MOESM1_ESM.pdf]

Questionnaire téléphonique sur l'utilisation de ressources de santé et la perte de productivité

ACCORD
Regroupement des forces pour gérer la douleur chronique

Instructions :

- Avant de débuter le questionnaire téléphonique, veuillez s.v.p. avoir devant vous TOUS les médicaments que vous utilisez actuellement.
- TOUTES les informations que nous vous demanderons aujourd'hui concernent les 3 DERNIERS MOIS → *Donc depuis* ________________________ *environ!*

ID patient :

☐☐☐ - ☐☐☐

Entrevue :

■ $_0$ T_0
☐ $_1$ T_1
☐ $_2$ T_2
☐ $_3$ T_3
☐ $_4$ T_4

Date :

☐☐ - ☐☐☐ - 20☐☐
Jour Mois (ex: JUN) Année

Interviewer :

☐ $_0$ A. Lacasse
☐ $_1$ ____________________

UTILISATION DE RESSOURCES DE SANTÉ

Instructions :
Les questions suivantes concernent les soins de santé utilisés :

- **AU COURS DES 3 DERNIERS MOIS**
- **À CAUSE DE VOTRE DOULEUR**.
- **POUR VOTRE SANTÉ SEULEMENT** et non pour la santé de votre conjoint ou de vos enfants.

1. HOSPITALISATIONS

Au cours des 3 derniers mois, avez-vous été hospitalisé(e) à cause de votre douleur? ☐1 Oui ☐0 Non

1.1. Si oui : Combien de fois avez-vous été hospitalisé(e)? Nombre de fois : ______

1.2. Si oui : Veuillez s.v.p. me préciser les dates de ces hospitalisations, la durée, les raisons, la distance approximative entre l'hôpital et votre foyer, le moyen de transport utilisé pour vous y rendre ainsi que les frais de stationnement déboursés s'il y a lieu :

DATE JOUR-MOIS-ANNÉE	DURÉE	RAISON(S)	DISTANCE DE VOTRE FOYER	MOYEN DE TRANSPORT EX: VOITURE, TAXI, AUTOBUS, ETC.	FRAIS DE STATIONNEMENT
………………	………jours	……………………	………km	………………………..	……………$
………………	………jours	……………………	………km	………………………..	……………$
………………	………jours	……………………	………km	………………………..	……………$
………………	………jours	……………………	………km	………………………..	……………$
………………	………jours	……………………	………km	………………………..	……………$
………………	………jours	……………………	………km	………………………..	……………$
………………	………jours	……………………	………km	………………………..	……………$

2. VISITES À L'URGENCE

<u>Au cours des 3 derniers mois</u>, avez-vous eu des visites à l'urgence à cause de votre douleur? ☐1 Oui ☐0 Non

2.1. Si oui : Combien de fois avez-vous été à l'urgence? Nombre de fois : ______

2.2. Si oui : Veuillez s.v.p. me préciser les dates de ces visites, la durée, les raisons, la distance approximative entre l'urgence et votre foyer, le moyen de transport utilisé pour vous y rendre ainsi que les frais de stationnement déboursés s'il y a lieu :

**DATE** JOUR-MOIS-ANNÉE	**DURÉE**	**RAISON(S)**	**DISTANCE DE VOTRE FOYER**	**MOYEN DE TRANSPORT** EX: VOITURE, TAXI, AUTOBUS, ETC.	**FRAIS DE STATIONNEMENT**
………………	………jours	……………………	……………km	………………………..	……………$
………………	………jours	……………………	……………km	………………………..	……………$
………………	………jours	……………………	……………km	………………………..	……………$
………………	………jours	……………………	……………km	………………………..	……………$
………………	………jours	……………………	……………km	………………………..	……………$

3. VISITES CHEZ LE MÉDECIN

<u>Au cours des 3 derniers mois</u>, avez-vous consulté un médecin à cause de votre douleur? ☐[1] Oui ☐[0] Non

3.1. Si oui : Combien de visites avez-vous eues chez les différents médecins suivants :

- Médecin de famille (omnipraticien)	Nombre de fois : _____
- Anesthésiste	Nombre de fois : _____
- Neurochirurgien	Nombre de fois : _____
- Neurologue	Nombre de fois : _____
- Orthopédiste	Nombre de fois : _____
- Physiatre	Nombre de fois : _____
- Psychiatre	Nombre de fois : _____
- Radiologiste	Nombre de fois : _____
- Rhumatologue	Nombre de fois : _____
- Clinique de la douleur	Nombre de fois : _____
- Autre : ____________________________________	Nombre de fois : _____

3.2. Si oui : Veuillez s.v.p. me préciser les dates, le type de médecin rencontré, les raisons de ces visites, la distance approximative entre les cliniques médicales et votre foyer, le moyen de transport utilisé pour vous y rendre ainsi que les frais de stationnement déboursés s'il y a lieu :

**DATE** JOUR-MOIS-ANNÉE	**TYPE DE MÉDECIN**	**RAISON(S)**	**DISTANCE DE VOTRE FOYER**	**MOYEN DE TRANSPORT** EX : VOITURE, TAXI, AUTOBUS, ETC.	**FRAIS DE STATIONNEMENT**
…………………	……………………	……………………	……………km	………………………..	……………$
…………………	……………………	……………………	……………km	………………………..	……………$
…………………	……………………	……………………	……………km	………………………..	……………$
…………………	……………………	……………………	……………km	………………………..	……………$
…………………	……………………	……………………	……………km	………………………..	……………$

4. VISITES CHEZ UN AUTRE PROFESSIONNEL DE LA SANTÉ

Au cours des 3 derniers mois, avez-vous consulté un autre professionnel de la santé à cause de votre douleur? ☐$_1$ Oui ☐$_0$ Non

4.1. Si oui : Combien de visites avez-vous eues chez ces différents professionnels :

- Infirmière	Nombre de fois : ______
- Acupuncteur	Nombre de fois : ______
- Art-thérapeute	Nombre de fois : ______
- Chiropraticien	Nombre de fois : ______
- Ergothérapeute	Nombre de fois : ______
- Homéopathe	Nombre de fois : ______
- Hypnothérapeute	Nombre de fois : ______
- Kinésiologue / Kinésiothérapeute	Nombre de fois : ______
- Massothérapeute	Nombre de fois : ______
- Naturopathe	Nombre de fois : ______
- Ostéopathe	Nombre de fois : ______
- Pharmacien	Nombre de fois : ______
- Physiothérapeute	Nombre de fois : ______
- Psychologue	Nombre de fois : ______
- Travailleur social	Nombre de fois : ______
- Autre : ________________________________	Nombre de fois : ______

(SUITE)

4.2. Si oui : Veuillez s.v.p. me préciser les dates, le type de professionnel rencontré, les raisons de ces visites, la distance entre le lieu de la visite et votre foyer, le moyen de transport utilisé pour vous y rendre, les frais de stationnement déboursés ainsi que le remboursement de ces consultations s'il y a lieu :

**DATE**<br>JOUR-MOIS-ANNÉE	**TYPE DE PROFESSIONNEL**	**RAISON(S)**	**DISTANCE DE VOTRE FOYER**	**MOYEN DE TRANSPORT**<br>EX: VOITURE, TAXI, AUTOBUS, ETC.	**FRAIS DE STATIONNEMENT**	**MONTANT PAYÉ POUR LES SOINS**	**ALLEZ-VOUS ÊTRE REMBOURSÉ(E)?**	**SI OUI, % <u>OU</u> MONTANT REMBOURSÉ?**	**SOURCE DU REMBOURSEMENT?**<br>EX: RAMQ, ASSUREUR PRIVÉ, CSST, ETC.
……………………	………………	………………	…………km	……………………………	……………$	…………$	☐Oui ☐Non	……………	…………………………
……………………	………………	………………	…………km	……………………………	……………$	…………$	☐Oui ☐Non	……………	…………………………
……………………	………………	………………	…………km	…………………………	……………$	…………$	☐Oui ☐Non	……………	…………………………
……………………	………………	………………	…………km	……………………………	……………$	…………$	☐Oui ☐Non	……………	…………………………
……………………	………………	………………	…………km	……………………………	……………$	…………$	☐Oui ☐Non	……………	…………………………
……………………	………………	………………	…………km	……………………………	……………$	…………$	☐Oui ☐Non	……………	…………………………
……………………	………………	………………	…………km	…………………………	……………$	…………$	☐Oui ☐Non	……………	…………………………
……………………	………………	………………	…………km	……………………………	……………$	…………$	☐Oui ☐Non	……………	…………………………
……………………	………………	………………	…………km	……………………………	……………$	…………$	☐Oui ☐Non	……………	…………………………
……………………	………………	………………	…………km	……………………………	……………$	…………$	☐Oui ☐Non	……………	…………………………
……………………	………………	………………	…………km	…………………………	……………$	…………$	☐Oui ☐Non	……………	…………………………
……………………	………………	………………	…………km	……………………………	……………$	…………$	☐Oui ☐Non	……………	…………………………

INTERVENTIONS POUR VOTRE DOULEUR

1. **<u>Au cours des 3 derniers mois</u>**, est-ce que vous avez subi une intervention afin de soulager/traiter **VOTRE DOULEUR**?

   Exemples : Infiltration, Bloc, Chirurgie, Implant d'une pompe, Péridurale, etc.

   ☐1 Oui ☐0 Non

1.1. Si oui, précisez :

**Date** JOUR-MOIS-ANNÉE	**Type d'intervention**
……………………..	………………………………………………………………
……………………..	………………………………………………………………
……………………..	………………………………………………………………
……………………..	………………………………………………………………
……………………..	………………………………………………………………
……………………..	………………………………………………………………
……………………..	………………………………………………………………
……………………..	………………………………………………………………

Injection épidurale
Infusion épidurale continue
Bloc caudal/épidurale caudale
Injection intradiscale
Bloc facettaire
Bloc branche médiane
Bloc foraminal
Bloc paravertébral
Bloc du nerf d'Arnold
Bloc d'un nerf intercostal
Bloc d'un nerf périphérique
Injection de point gachette
Injection intra-articulaire
Bloc régional intraveineux/bloc veineux
Injection de toxine botulinique
Injection intrathécale
Infusion intrathécale continue
Neurostimulation moelle épinière
Bloc sympathique (anesthésique local)
stellaire
coeliaque
lombaire
ganglion impar
Bloc sympathique (agent neurolytique)
stellaire
coeliaque
lombaire
ganglion impar
Neurolyse intrathécale
Neurolyse branche médiane
Neurolyse nerf périphérique
IDET
Coblation
Infiltration subcicatricielle
Infiltration musculaire
Perfusion zylocaïne

ACHAT DE MÉDICAMENTS EN VENTE LIBRE POUR VOTRE DOULEUR

1. **<u>Au cours des 3 derniers mois</u>**, est-ce que vous <u>ou</u> un membre de votre famille avez acheté des médicaments **en vente libre** afin de soulager/traiter **VOTRE DOULEUR**? *Exemples : Advil®, Motrin®, Tylenol®, etc.*

☐1 Oui ☐0 Non

ATTENTION : Médicaments qui ne nécessitent pas la prescription d'un médecin lors de l'achat.

1.1. Si oui, précisez :

**Date** Jour-Mois-Année	**Nom du médicament pour votre douleur**	**Coût total incluant les taxes**	**NB d'achats depuis les 3 derniers mois**	**Allez-vous être remboursé(e)?**	**Si oui, % <u>ou</u> montant remboursé?**	**Source du remboursement?** Ex: RAMQ, assureur privé, CSST, etc.	**Utilisé actuellement?**	**Dose**	**Unités**	**Fréquence**
………………	……………………..	………..$	…… x	☐Oui ☐Non	…………..	………………	☐Oui ☐Non		☐0 mg ☐1 mcg ☐2 ui	___ fois par: ☐jr ☐sem ☐mois
………………	……………………..	………..$	…… x	☐Oui ☐Non	…………..	………………	☐Oui ☐Non		☐0 mg ☐1 mcg ☐2 ui	___ fois par: ☐jr ☐sem ☐mois
………………	……………………..	………..$	…… x	☐Oui ☐Non	…………..	………………	☐Oui ☐Non		☐0 mg ☐1 mcg ☐2 ui	___ fois par: ☐jr ☐sem ☐mois
………………	……………………..	………..$	…… x	☐Oui ☐Non	…………..	………………	☐Oui ☐Non		☐0 mg ☐1 mcg ☐2 ui	___ fois par: ☐jr ☐sem ☐mois
………………	……………………..	………..$	…… x	☐Oui ☐Non	…………..	………………	☐Oui ☐Non		☐0 mg ☐1 mcg ☐2 ui	___ fois par: ☐jr ☐sem ☐mois

2. [*Mis à part ce qui est mentionné ci-haut* (les nommer si applicable)] Utilisez-vous actuellement des médicaments **en vente libre** afin de soulager/traiter **VOTRE DOULEUR?**

☐₁ Oui ☐₀ Non

2.1. Si oui, précisez :

**Date** Jour-Mois-Année	**Nom du médicament pour votre douleur**	**Coût total incluant les taxes**	**NB d'achats depuis la dernière entrevue**	**Utilisé actuellement?**	**Dose**	**Unités**	**Fréquence**
………………	……………………..	**0 $**	**0 x**	☐Oui ☐Non		☐0 mg ☐1 mcg ☐2 ui	____ fois par: ☐jr ☐sem ☐mois
………………	……………………..	**0 $**	**0 x**	☐Oui ☐Non		☐0 mg ☐1 mcg ☐2 ui	____ fois par: ☐jr ☐sem ☐mois
………………	……………………..	**0 $**	**0 x**	☐Oui ☐Non		☐0 mg ☐1 mcg ☐2 ui	____ fois par: ☐jr ☐sem ☐mois
………………	……………………..	**0 $**	**0 x**	☐Oui ☐Non		☐0 mg ☐1 mcg ☐2 ui	____ fois par: ☐jr ☐sem ☐mois
………………	……………………..	**0 $**	**0 x**	☐Oui ☐Non		☐0 mg ☐1 mcg ☐2 ui	____ fois par: ☐jr ☐sem ☐mois
………………	……………………..	**0 $**	**0 x**	☐Oui ☐Non		☐0 mg ☐1 mcg ☐2 ui	____ fois par: ☐jr ☐sem ☐mois

ACHAT DE MÉDICAMENTS PRESCRITS POUR VOTRE DOULEUR

1. **<u>Au cours des 3 derniers mois</u>**, est-ce que vous <u>ou</u> un membre de votre famille avez acheté des **médicaments prescrits** afin de soulager/traiter **VOTRE DOULEUR**?

   Exemples : Naproxène, Amitriptyline (Elavil®), Pregabaline (Lyrica®), etc.
   ☐1 Oui ☐0 Non

ATTENTION : Certains médicaments tels que les <u>antidépresseurs</u> et les <u>anticonvulsivants</u> peuvent êtres prescrits pour la douleur.

Si le sujet a acheté un médicament de ce genre, DEMANDER s'il souffre de dépression ou d'épilepsie?

NE PAS INSCRIRE les médicaments <u>non reliés</u> à la douleur.
Exemples : Antidépresseur pour la dépression uniquement OU Anticonvulsivant pour l'épilepsie uniquement.

Si le patient ne sait pas si son médicament est pour la dépression ou la douleur l'INSCRIRE.

1.1. Si oui, précisez :

**Date** Jour-Mois-Année	**Nom du médicament pour votre douleur**	**NB d'achats depuis les 3 derniers mois**	**Allez-vous être remboursé(e)?**	**Si oui, % <u>ou</u> montant remboursé?**	**Source du remboursement?** Ex: RAMQ, assureur privé, CSST, etc.	**Utilisé actuellement?**	**Dose**	**Unités**	**Fréquence**
………………	……………………..	………… **x**	☐Oui ☐Non	…………..	………………	☐Oui ☐Non		☐0 mg ☐1 mcg ☐2 ui	____ fois par: ☐jr ☐sem ☐mois
………………	……………………..	………… **x**	☐Oui ☐Non	…………..	………………	☐Oui ☐Non		☐0 mg ☐1 mcg ☐2 ui	____ fois par: ☐jr ☐sem ☐mois
………………	……………………..	………… **x**	☐Oui ☐Non	…………..	………………	☐Oui ☐Non		☐0 mg ☐1 mcg ☐2 ui	____ fois par: ☐jr ☐sem ☐mois

(suite)

**Date** Jour-Mois-Année	**Nom du médicament pour votre douleur**	**NB d'achats depuis les 3 derniers mois**	**Allez-vous être remboursé(e)?**	**Si oui, % ou montant remboursé?**	**Source du remboursement?** Ex: RAMQ, assureur privé, CSST, etc.	**Utilisé actuellement?**	**Dose**	**Unités**	**Fréquence**
………………	……………………..	………… **x**	☐Oui ☐Non	…………..	………………	☐Oui ☐Non		☐0 mg ☐1 mcg ☐2 ui	____ fois par: ☐jr ☐sem ☐mois
………………	……………………..	………… **x**	☐Oui ☐Non	…………..	………………	☐Oui ☐Non		☐0 mg ☐1 mcg ☐2 ui	____ fois par: ☐jr ☐sem ☐mois
………………	……………………..	………… **x**	☐Oui ☐Non	…………..	………………	☐Oui ☐Non		☐0 mg ☐1 mcg ☐2 ui	____ fois par: ☐jr ☐sem ☐mois
………………	……………………..	………… **x**	☐Oui ☐Non	…………..	………………	☐Oui ☐Non		☐0 mg ☐1 mcg ☐2 ui	____ fois par: ☐jr ☐sem ☐mois
………………	……………………..	………… **x**	☐Oui ☐Non	…………..	………………	☐Oui ☐Non		☐0 mg ☐1 mcg ☐2 ui	____ fois par: ☐jr ☐sem ☐mois
………………	……………………..	………… **x**	☐Oui ☐Non	…………..	………………	☐Oui ☐Non		☐0 mg ☐1 mcg ☐2 ui	____ fois par: ☐jr ☐sem ☐mois
………………	……………………..	………… **x**	☐Oui ☐Non	…………..	………………	☐Oui ☐Non		☐0 mg ☐1 mcg ☐2 ui	____ fois par: ☐jr ☐sem ☐mois
………………	……………………..	………… **x**	☐Oui ☐Non	…………..	………………	☐Oui ☐Non		☐0 mg ☐1 mcg ☐2 ui	____ fois par: ☐jr ☐sem ☐mois

1.2. Si oui, <u>au cours des 3 derniers mois</u>, est-ce que vous <u>ou</u> un membre de votre famille avez acheté des **médicaments prescrits <u>afin de soulager les effets indésirables des médicaments pour</u> VOTRE DOULEUR**?

Exemples : Antiacides, laxatifs… : Senokot®, Nexium®, Prevacid®, Losec®, Pantoloc®, Pepcid®, Zantac®, etc.

ATTENTION : Ne pas inscrire les médicaments <u>non reliés</u> à la prise de médicaments pour la douleur.

Exemple : Laxatif pour un problème de constipation chronique.

**Date** Jour-Mois-Année	**Nom du médicament pour votre douleur**	**NB d'achats depuis les 3 derniers mois**	**Allez-vous être remboursé(e)?**	**Si oui, % <u>ou</u> montant remboursé?**	**Source du remboursement?** Ex: RAMQ, assureur privé, CSST, etc.	**Utilisé actuellement?**	**Dose**	**Unités**	**Fréquence**
………………	……………………	………… **x**	☐Oui ☐Non	…………..	………………	☐Oui ☐Non		☐0 mg ☐1 mcg ☐2 ui	___ fois par: ☐jr ☐sem ☐mois
………………	……………………	………… **x**	☐Oui ☐Non	…………..	………………	☐Oui ☐Non		☐0 mg ☐1 mcg ☐2 ui	___ fois par: ☐jr ☐sem ☐mois
………………	……………………	………… **x**	☐Oui ☐Non	…………..	………………	☐Oui ☐Non		☐0 mg ☐1 mcg ☐2 ui	___ fois par: ☐jr ☐sem ☐mois
………………	……………………	………… **x**	☐Oui ☐Non	…………..	………………	☐Oui ☐Non		☐0 mg ☐1 mcg ☐2 ui	___ fois par: ☐jr ☐sem ☐mois
………………	……………………	………… **x**	☐Oui ☐Non	…………..	………………	☐Oui ☐Non		☐0 mg ☐1 mcg ☐2 ui	___ fois par: ☐jr ☐sem ☐mois

ACHAT DE PRODUITS NATURELS POUR VOTRE DOULEUR

1. **<u>Au cours des 3 derniers mois</u>**, est-ce que vous <u>ou</u> un membre de votre famille avez acheté des **produits naturels** afin de soulager/traiter **VOTRE DOULEUR**?

   Exemples : Cannabis, Haschich, Herbes, Produits homéopathiques, Baume du tigre® etc.

   ☐1 Oui ☐0 Non

1.1. Si oui, précisez :

**Date** *Jour-Mois-Année*	**Nom du produit naturel pour votre douleur**	**Coût total incluant les taxes**
....	....	...............$
....	....	...............$
....	....	...............$
....	....	...............$
....	....	...............$
....	....	...............$
....	....	...............$
....	....	...............$
....	....	...............$
....	....	...............$

2. [*Mis à part ce qui est mentionné ci-haut* (les nommer si applicable)] Utilisez-vous actuellement des **produits naturels** afin de soulager/traiter **VOTRE DOULEUR?**

☐1 Oui ☐0 Non

2.1. Si oui, précisez :

**Date** Jour-Mois-Année	**Nom du produit naturel pour votre douleur**	**Coût total incluant les taxes**	**Utilisé actuellement?**
………………	………………………………………..	**0 $**	☐Oui ☐Non
………………	………………………………………..	**0 $**	☐Oui ☐Non
………………	………………………………………..	**0 $**	☐Oui ☐Non
………………	………………………………………..	**0 $**	☐Oui ☐Non
………………	………………………………………..	**0 $**	☐Oui ☐Non
………………	………………………………………..	**0 $**	☐Oui ☐Non

ACHAT DE FOURNITURES POUR VOTRE DOULEUR

1. **<u>Au cours des 3 derniers mois</u>**, est-ce que vous <u>ou</u> un membre de votre famille avez acheté des **fournitures** afin de soulager/traiter **VOTRE DOULEUR**?

   Exemples : Support lombaire, Béquilles, Chaise roulante, Sac Magique® etc.
   ☐1 Oui ☐0 Non

1.1. Si oui, précisez :

**Date** *Jour-Mois-Année*	**Nom de la fourniture pour votre douleur**	**Coût total incluant les taxes**	**Allez-vous être remboursé(e)?**	**Si oui, % <u>ou</u> montant remboursé?**	**Source du remboursement?** Ex: RAMQ, assureur privé, CSST, etc.
………………	………………………..	…………..$	☐Oui ☐Non	………………..	………………………....
………………	………………………..	…………..$	☐Oui ☐Non	………………..	………………………....
………………	………………………..	…………..$	☐Oui ☐Non	………………..	………………………....
………………	………………………....	…………..$	☐Oui ☐Non	………………..	………………………....
………………	………………………....	…………..$	☐Oui ☐Non	………………..	………………………....
………………	………………………....	…………..$	☐Oui ☐Non	………………..	………………………....
………………	………………………..	…………..$	☐Oui ☐Non	………………..	………………………....
………………	………………………..	…………..$	☐Oui ☐Non	………………..	………………………....
………………	………………………..	…………..$	☐Oui ☐Non	………………..	………………………....
………………	………………………..	…………..$	☐Oui ☐Non	………………..	………………………....

EFFETS INDÉSIRABLES

1. Maintenant, nous allons discuter des effets indésirables que vous auriez pu avoir **<u>À CAUSE</u> DE VOTRE TRAITEMENT ACTUEL CONTRE LA DOULEUR** (médicaments analgésiques ou produits naturels)?

☐$_0$ Non applicable, car aucun médicament, cannabis ou produit naturel n'a été utilisé pour soulager la douleur.

ATTENTION : Ne pas inscrire les effets indésirables <u>non reliés</u> au traitement de la douleur.

Exemples : Gain de poids relié à la cessation tabagique,
Vomissements reliés à une intoxication alimentaire.

a) Vertige / étourdissement?	☐$_0$ aucun	☐$_1$léger	☐$_2$ modéré	☐$_3$ sévère
b) Somnolence ?	☐$_0$ aucun	☐$_1$léger	☐$_2$ modéré	☐$_3$sévère
c) Confusion ?	☐$_0$ aucun	☐$_1$léger	☐$_2$ modéré	☐$_3$sévère
d) Nausée ?	☐$_0$ aucun	☐$_1$léger	☐$_2$ modéré	☐$_3$sévère
e) Vomissement?	☐$_0$ aucun	☐$_1$léger	☐$_2$ modéré	☐$_3$sévère
f) Perte de mémoire ?	☐$_0$ aucun	☐$_1$ léger	☐$_2$ modéré	☐$_3$sévère
g) Bouche sèche ?	☐$_0$ aucun	☐$_1$ léger	☐$_2$modéré	☐$_3$sévère
h) Démangeaison ?	☐$_0$ aucun	☐$_1$ léger	☐$_2$ modéré	☐$_3$sévère
i) Inconfort abdominal ?	☐$_0$ aucun	☐$_1$ léger	☐$_2$ modéré	☐$_3$ sévère
j) Constipation ?	☐$_0$ aucun	☐$_1$ léger	☐$_2$ modéré	☐$_3$sévère
k) Ralentissement du débit urinaire ?	☐$_0$ aucun	☐$_1$ léger	☐$_2$ modéré	☐$_3$ sévère
l) Fatigue ?	☐$_0$ aucun	☐$_1$ léger	☐$_2$ modéré	☐$_3$sévère
m) Insomnie ?	☐$_0$ aucun	☐$_1$ léger	☐$_2$ modéré	☐$_3$sévère
n) Enflure ?	☐$_0$ aucun	☐$_1$ léger	☐$_2$ modéré	☐$_3$ sévère
o) Gain de poids ?	☐$_0$ aucun	☐$_1$ léger	☐$_2$ modéré	☐$_3$ sévère
p) Vision brouillée ?	☐$_0$ aucun	☐$_1$ léger	☐$_2$ modéré	☐$_3$ sévère
q) Baisse de libido?	☐$_0$ aucun	☐$_1$léger	☐$_2$ modéré	☐$_3$sévère
r) Hallucinations?	☐$_0$ aucun	☐$_1$ léger	☐$_2$ modéré	☐$_3$sévère
s) Cauchemars?	☐$_0$ aucun	☐$_1$ léger	☐$_2$ modéré	☐$_3$sévère
t) Autres : (spécifiez) ___________	☐$_0$ aucun	☐$_1$ léger	☐$_2$ modéré	☐$_3$ sévère
u) Autres : (spécifiez) ___________	☐$_0$ aucun	☐$_1$ léger	☐$_2$ modéré	☐$_3$ sévère

Toujours en raison de votre traitement contre la douleur!

AIDE DOMESTIQUE RÉMUNÉRÉ

1. **<u>Au cours des 3 derniers mois,</u>** avez-vous rémunéré d'autres personnes (membre de la famille, ami, voisin, etc.) pour vous aider avec les tâches ménagères ou familiales **À CAUSE DE VOTRE DOULEUR?**

   Même si cette rémunération n'est pas déclarée, soyez certains que cette information demeurera confidentielle!

OUI	NON
☐1	☐0

   1.1. Le cas échéant, veuillez s.v.p. me préciser les dates, le nombre d'heures d'aide avec les tâches ménagères ou familiales, le montant total payé par vous ainsi que le type d'aide apporté :

DATE *JOUR-MOIS-ANNÉE*	TOTAL D'HEURES	MONTANT TOTAL PAYÉ	TYPE D'AIDE APPORTÉE
………………	……………hr	……………$	………………………
………………	……………hr	……………$	………………………
………………	……………hr	……………$	………………………
………………	……………hr	……………$	………………………
………………	……………hr	……………$	………………………
………………	……………hr	……………$	………………………
………………	……………hr	……………$	………………………
………………	……………hr	……………$	………………………
………………	……………hr	……………$	………………………
………………	……………hr	……………$	………………………

AUTRES FRAIS

1. Mis à part toutes les informations que vous avez fournies jusqu'à présent, avez-vous eu <u>d'autres frais</u> à assumer pour soigner **VOTRE DOULEUR** ou résultant de votre douleur **<u>au cours des 3 derniers mois</u>**? ☐1 Oui ☐0 Non

   Exemples : Dépenses payées par vos proches, rénovations, etc.

   1.1. Si oui, précisez :

DATE *JOUR-MOIS-ANNÉE*	TYPE DE FRAIS	MONTANT TOTAL PAYÉ
………………	…………………………………………………	……………$
………………	…………………………………………………	……………$
………………	…………………………………………………	……………$
………………	…………………………………………………	……………$
………………	…………………………………………………	……………$
………………	…………………………………………………	……………$

PERTE DE PRODUCTIVITÉ

1. **Au cours des 3 derniers mois**, aviez-vous un travail rémunéré?

☐$_1$ Oui ☐$_0$ Non

SI « **OUI**» ADMINISTRER LA SECTION **A** ET **B**

SI « **NON** » ADMINISTRER LA SECTION **B SEULEMENT**

SECTION A

1.1. Au cours des 3 derniers mois, combien de jours avez-vous été **absent(e**) de votre travail rémunéré en raison d'une **VISITE MÉDICALE RELIÉE AU TRAITEMENT DE VOTRE DOULEUR**?
Incluant la participation à une école interactionnelle, les rendez-vous à la clinique de la douleur, les hospitalisations, les visites à l'urgence, les visites chez un médecin, les visites chez un professionnel de la santé et les visites à la pharmacie, etc.

Nombre de journées: ___________

1.2. Au cours des 3 derniers mois, combien de jours avez-vous été **absent(e)** de votre travail rémunéré **À CAUSE DE VOTRE DOULEUR**?
Exemples : Vous aviez trop mal pour travailler, vous étiez inconfortable, vous êtes arrivé en retard, etc.

Nombre de journées: ___________

1.3. En moyenne, combien de jours par semaine êtes-vous normalement supposé travaillé?

Nombre de journées: ___________ /semaine

1 journée = 7.5 ou 8 heures

1.4. Grâce à votre travail rémunéré, quel était **VOTRE** revenu annuel avant impôts au cours des 3 derniers mois?

☐$_0$ Moins de 10 000 $
☐$_1$ Entre 10 000 $ et 19 999 $
☐$_2$ Entre 20 000 $ et 29 999 $
☐$_3$ Entre 30 000 $ et 39 999 $
☐$_4$ Entre 40 000 $ et 49 999 $
☐$_5$ Entre 50 000 $ et 59 999 $
☐$_6$ Entre 60 000 $ et 69 999 $
☐$_7$ Entre 70 000 $ et 79 999 $
☐$_8$ Entre 80 000 $ et 89 000 $
☐$_9$ Entre 90 000 $ et 99 000 $
☐$_{10}$ 100 000 $ et plus

☐ Ne désire pas répondre

SECTION B

Les questions suivantes portent sur le travail au foyer NON RÉMUNÉRÉ, incluant les tâches domestiques et celles liées aux soins des enfants et des proches.

1.5. Au cours des 3 derniers mois, combien de jours avez-vous été obligé(e) de cesser votre **travail au foyer non rémunéré** en raison d'une **VISITE MÉDICALE RELIÉE AU TRAITEMENT DE VOTRE DOULEUR**?
Incluant la participation à une école interactionnelle, les rendez-vous à la clinique de la douleur, les hospitalisations, les visites à l'urgence, les visites chez un médecin, les visites chez un professionnel de la santé et les visites à la pharmacie, etc.

Nombre de journées: ___________

1.6. Au cours des 3 derniers mois, combien de jours avez-vous été obligé(e) de cesser votre **travail au foyer non rémunéré À CAUSE DE VOTRE DOULEUR**?
Exemples : Vous aviez trop mal pour effectuer vos tâches, vous étiez inconfortable etc.

Nombre de journées: ___________

PROPENSITÉ À PAYER POUR L'INTERVENTION

Pour finir, une petite mise en situation…
Nous allons faire comme si vous aviez à payer pour participer aux Écoles Interactionnelles offertes dans votre région. Bien sûr, dans la réalité, votre participation présente ou future aux Écoles Interactionnelles est entièrement gratuite!

Compte tenu de vos symptômes douloureux, quel montant maximum seriez-vous prêt à payer pour participer à l'École interactionnelle de : _________$

LE QUESTIONNAIRE SE TERMINE ICI.

EST-CE QUE VOUS AVEZ DES QUESTIONS?

SI CE N'EST PAS DÉJÀ FAIT, JE VOUS INVITE ÉGALEMENT À RETOURNER VOTRE QUESTIONNAIRE PAPIER

JE VOUS REMERCIE BEAUCOUP DE VOTRE PARTICIPATION!

NOUS NOUS REPARLERONS DANS ENVIRON 2 MOIS POUR UNE ENTREVUE SIMILAIRE.
